# Editorial: Resilience and Health in the Chinese People During the COVID-19 Outbreak

**DOI:** 10.3389/fpsyt.2021.742960

**Published:** 2021-08-24

**Authors:** Julian Chuk-Ling Lai, Tina L. Rochelle

**Affiliations:** Department of Social and Behavioural Sciences, City University of Hong Kong, Kowloon, China

**Keywords:** COVID-19, resilience, mental health, coping, Chinese people

The COVID-19 pandemic is a global trauma. To date, the pandemic has not only taken away the lives of four million people, but also created an unprecedented impact on the mental health in both infected patients and non-infected populations, both directly due to the medical complications associated with infection, and indirectly because of the implementation of public health measures such as social distancing, lockdowns and quarantines [reviewed by Kontoangelos et al. (1) and Vindegaard and Benros (2)]. The availability of effective vaccines to the general public once sparked the hope of impending emergence from the trauma. Unfortunately, this has been undermined recently by the emergence of new and more contagious variants of the virus. Amidst the progressive return to “normal” in some places, a number of countries are now facing the challenges of a new wave of epidemic caused by the latest variant of the COVID-19 virus. Despite the pandemic's widespread impact on mental health in different populations including the general public and healthcare workers [e.g., (2)], the focus of research since the beginning of the outbreak has been on medical complications of infection. This collection is expected to fill this gap by focusing on the indirect or mental health impact of the COVID-19 pandemic, with special attention to the Chinese people during the early stage of the outbreak.

The editorial and call for submissions for a special edition on resilience and health of Chinese people during the Covid-19 outbreak received a great response. This special issue includes the papers and reports on the topic. In addition to evaluating the mental health impact of the outbreak, we are also interested in examining risk and resilience factors modulating the impact of stress related to the pandemic and mental health outcomes. The focus on China serves to highlight the importance of contextual factors in determining the impact and responses to the challenges of the pandemic at both the individual and collective level. This focus seems to be justified with hindsight because China is one of the very few economies emerging from this unprecedented global trauma (3). This recovery would not have taken place without the unique combination of strong leadership and collectivistic obedience (4, 5). Admittedly, the best that this collection can do is to provide a snapshot of the impact of and responses to the COVID-19 outbreak in China and other Chinese communities. Despite this limitation, it is hoped that the findings and ideas growing from this collection would be able to leave a inerasable mark in the timeline of psychiatric research.

This collection consists of 21 studies with a total of over 46,500 participants from different cities/provinces across China. A number of studies used a nationwide sample from various cities or provinces (e.g., Bressington et al.; Chen et al.). A diversified array of mental health outcomes including depression (e.g., Bressington et al.; Zhang Y-t. et al.), anxiety (e.g., Chen et al.), symptoms of PTSD (e.g., Duan et al.), perceived stress (e.g., Zhang X. et al.), and psychosomatic burden (e.g., Yi et al.) were examined, and in a handful of studies, in relation to specific stressors (e.g., Wong et al.). In addition to risk factors, factors that confer resilience to stressful situations like hope (e.g., Zhang Z. et al.), gratitude (e.g., Tong and Oh), adaptive coping (e.g., Cheng et al.), and tolerance of uncertainty (e.g., He et al.) were also examined. Gender differences in vulnerability were examined in Liu et al., which revealed heightened vulnerability of post-traumatic stress and depression among younger men aged 26–30 years. Public health policy recommendations to alleviate the “emotional shocks” and psychiatric aftermaths of the outbreak were also put forward in specific studies (e.g., Zhao et al.). The findings from the studies featured in this special issue echo the wider health and psychology literature emphasizing the importance of resilience and adaptability in the move forward with COVID. Within the Chinese context, psychologists and behavioral scientists have provided major contributions in the effort to raise awareness, educate and reduce the impact of COVID-19. The studies featured within this special issue have identified key areas, issues and factors that could be targeted in interventions. However, much less is known about what types of interventions are effective, for what types of patient groups and populations etc. In the move forward with COVID, this must now be the next step in enlightening our knowledge.

## Author Contributions

All authors listed have made a substantial, direct and intellectual contribution to the work, and approved it for publication.

## Conflict of Interest

The authors declare that the research was conducted in the absence of any commercial or financial relationships that could be construed as a potential conflict of interest.

## Publisher's Note

All claims expressed in this article are solely those of the authors and do not necessarily represent those of their affiliated organizations, or those of the publisher, the editors and the reviewers. Any product that may be evaluated in this article, or claim that may be made by its manufacturer, is not guaranteed or endorsed by the publisher.
